# Preliminary investigation of nutritional intake among female university students using the brief-type self-administered diet history questionnaire (BDHQ)

**DOI:** 10.20407/fmj.2025-031

**Published:** 2026-05-14

**Authors:** Masato Sugiura, Harumi Kato, Keisuke Iwase, Hironori Tsuzuki, Qiongai Jin, Yumi Akashi, Mariko Umemura, Noriko Suzuki, Tomoko Tanaka, Yukie Kitamura, Yuki Higashimoto, Iku Fujiwara, Naoki Yamamoto, Mikiko Shimizu

**Affiliations:** 1 Faculty of Nursing, Fujita Health University, School of Health Sciences, Toyoake, Aichi, Japan; 2 Department of Nursing, Toyohashi Sozo University, Toyohashi, Aichi, Japan; 3 Department of Nursing Science, Kitasato University School of Health Science, Minamiuonuma, Niigata, Japan; 4 Faculty of Health Care and Nursing, Juntendo University, Urayasu, Chiba, Japan; 5 Department of Nursing, School of Nursing, Tokyo University of Information Sciences, Chiba, Chiba, Japan; 6 Department of Clinical Microbiology, Fujita Health University, School of Medical Sciences, Toyoake, Aichi, Japan; 7 Research Promotion Headquarters, Fujita Health University, Toyoake, Aichi, Japan

**Keywords:** Female university students, Brief-type self-administered diet history questionnaire (BDHQ), Eating habits, Nutritional intake, Estimated energy requirements

## Abstract

**Objective:**

This study examines the dietary intake and eating habits of female university students and compares key nutritional indicators according to the body mass index (BMI) categories and adequacy of estimated energy requirements.

**Methods:**

Dietary surveys were conducted among 50 female nursing students using the brief-type self-administered diet history questionnaire (BDHQ). Participants were classified into BMI categories (underweight group, n=7; normal-weight group, n=41) and energy-intake adequacy groups based on estimated energy requirements (inadequate energy intake group, n=43; adequate/excess energy intake group, n=7). Nutrient intake status was examined between energy intake adequacy groups, with additional consideration of the BMI categories.

**Results:**

No significant differences in energy or macronutrient intake were observed between BMI-based groups (p>0.05). In contrast, when comparing energy intake adequacy, 88.4% of the participants in the inadequate energy intake group had insufficient carbohydrate intake, and their intakes of protein, vitamin C, iron, and folate were lower than those in the adequate/excess energy intake group (all p<0.05).

**Discussion:**

Among female university students, no group differences were detected based on BMI alone. However, in the inadequate energy intake group, marked inadequacies were observed in the intake of nutrients. These findings indicate that dietary problems cannot be captured solely by BMI and require individualized nutritional counseling.

**Conclusion:**

Nutritional assessment of female university students should incorporate BMI and adequacy of energy intake relative to estimated energy requirements, with individualized approaches tailored to each student.

## Introduction

The proportion of underweight women aged 20–29 years in Japan is as high as 20.2%.^1^ This high prevalence has been attributed to a strong preference for thinness, frequent dieting experiences, and changes in exercise habits and body weight perception.^2^ Dietary habits differ depending on whether women desire to be thinner, and approximately 90% of underweight women have insufficient nutrient intake, with particularly marked deficiencies in folate and vitamin B_12_.^3^ Such nutritional deficiencies, combined with a lack of well-balanced dietary habits before pregnancy, may adversely affect maternal health and pregnancy outcomes.^4^ Therefore, improving the nutritional status of young women from an early stage is an important public health issue.

In particular, female university students constitute a high-risk group for being underweight, as economic and time constraints, limited nutrition knowledge, and a strong desire for thinness often overlap, resulting in low intake of carbohydrates and essential nutrients.^5^ The importance of preconception health management (preconception care) for this generation has been internationally recognized.^6,7^ Recent large-scale studies on Japanese university students have reported sex-specific differences and trends in dietary intake patterns according to body mass index (BMI) categories.^8^ However, substantial inter-individual variability suggests that BMI classification alone does not necessarily capture differences in nutrient intake among female students. The impact of diverse living backgrounds and differences in estimated energy requirements on nutritional status has not been sufficiently examined.

In this context, this study aimed to examine the dietary intake and eating habits of female university students and analyze key nutritional indicators by BMI categories and estimated energy requirement adequacy. The main results are presented according to groups defined by energy intake adequacy relative to the estimated energy requirements, as no significant differences were observed in the comparisons based on BMI categories.

## Methods

### Study design and participants

This cross-sectional observational study targeted approximately 500 female nursing students in grades 1–4 enrolled in the Department of Nursing at a private university (A University) in Aichi Prefecture between December 2022 and February 2023. Participants were recruited through a campus bulletin, and 53 students who responded were provided with an explanation of the study, and they gave written and oral informed consent. A brief-type self-administered diet history questionnaire (BDHQ) was administered to assess the dietary habits during the preceding month. The questionnaire was self-administered, and body mass index (BMI) was calculated from the self-reported height and weight recorded at the time of BDHQ completion.

All collected data were aggregated by a research contractor and then organized in Excel; responses with missing values or clearly implausible entries were excluded, leaving 50 participants for analysis. BMI categories were defined according to the Guidelines for the Management of Obesity Disease (2022) issued by the Japan Society for the Study of Obesity: underweight, BMI<18.5; normal weight, 18.5≤BMI<25.0; and obesity grade 1, 25.0≤BMI<30.0.^9^ Additionally, those with obesity grade 1 were excluded from analyses comparing BMI-based groups (underweight versus normal weight) because participants with BMI≥25.0 were classified as obese and were expected to have markedly different dietary intake patterns compared with underweight and normal-weight individuals. In contrast, all participants, including those with obesity grade 1, were included in the analyses that compared the inadequate energy intake group with the adequate/excess energy intake group.

### Brief-type self-administered diet history questionnaire (BDHQ)

The BDHQ used in this study is a four-page (A3, double-sided) questionnaire that can be completed in approximately 15 minutes and is designed to conveniently assess dietary habits in the previous month. Developed as a simplified version of the comprehensive Diet History Questionnaire (DHQ), it consisted of approximately 80 questions covering 58 food items and enabled the estimation of energy and nutrient intakes, food group intakes, and qualitative dietary behavior indicators.^10^ The BDHQ has been widely used in large-scale epidemiological studies and health guidance for Japanese adults, and its utility for reasonably assessing dietary intake has been demonstrated, although the accuracy of estimates for some nutrients, such as salt intake, is limited.^10,11^

### Statistical analysis

For each group, median values and interquartile ranges (IQR) were calculated for energy intake; the three major macronutrients (protein, fat, and carbohydrates); minerals (sodium, potassium, calcium, magnesium, iron, zinc, and copper); vitamins (vitamin D, vitamin B_1_, vitamin B_12_, vitamin C, and folate); and other components (saturated fatty acids, monounsaturated fatty acids, polyunsaturated fatty acids, total dietary fiber, salt equivalent, and sucrose). Differences between the underweight and normal-weight groups, defined by BMI, were examined using the Mann–Whitney U test. The same test was used to compare the inadequate energy intake group with the adequate/excess energy intake group.

For macronutrients, reference values were set at 65 g/day for protein, 50 g/day for fat, and 250 g/day for carbohydrates based on the Dietary Reference Intakes for Japanese (2020 edition), and tendencies were evaluated according to whether intakes were below or above these reference values.^12^ Statistical analyses were conducted using IBM SPSS, and differences between the two groups were considered statistically significant at p<0.05.

### Ethical considerations

This study was approved by the Ethics Review Committee of the Fujita Health University (approval number: HM18-156). The participants received written and oral explanations regarding the study purpose and procedures, voluntary participation, protection of personal information, and dissemination of results, and written informed consent was obtained. Participation was voluntary and participants were informed that they could withdraw their consent at any time. All data were anonymized and used solely for research purposes.

## Results

### Participant characteristics and group classification

**Figure 1:**
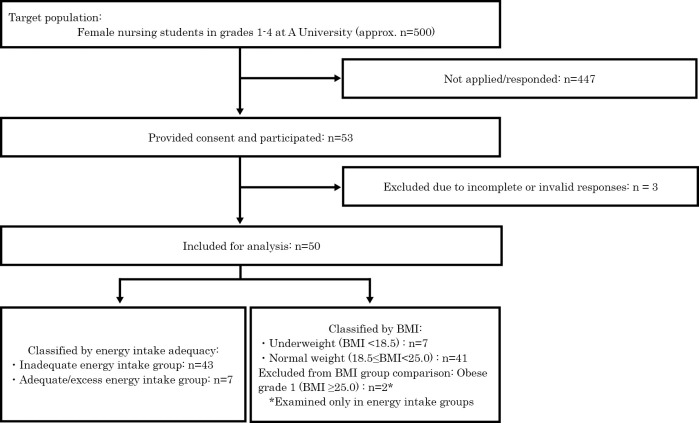
Flow diagram of participant recruitment, eligibility assessment, exclusion, grouping, and analysis Approximately 500 female nursing students (grades 1–4) were invited at A University, 53 consented to participate. After excluding three with incomplete/invalid responses, 50 patients were analyzed. The participants were classified according to energy intake adequacy (inadequate: n=43; adequate/excess: n=7) and BMI (underweight: n=7; normal weight: n=41). Two participants with grade 1 obesity (BMI≥25.0) were excluded from BMI comparisons but included in energy intake analyses.

The flow diagram of participant recruitment and inclusion is shown in Figure 1. Among the 50 participants included in the analysis, participants’ age ranged from 18 to 22 years (median 19.0 years [IQR 19.0–21.0]), height ranged from 148.8 to 171.5 cm (median 158.0 cm [IQR 154.0–162.0]), weight ranged from 38.5 to 68.0 kg (median 52.5 kg [IQR 47.6–56.1]), and BMI ranged from 16.2 to 27.0 kg/m^2^ (median 20.6 kg/m^2^ [IQR 19.4–21.9]). Table 1 presents the baseline characteristics according to energy intake adequacy, with 43 participants in the inadequate energy intake group and seven in the adequate/excess energy intake group. There were no statistically significant differences between the two groups in terms of age, height, weight, or BMI (p>0.05).

### Estimated energy requirements and nutrient intake

Table 2 shows the nutritional intake status of the inadequate (n=43) and adequate/excess energy intake groups (n=7). In the inadequate energy intake group, 37 participants (86.0%) consumed less than 65 g/day of protein, 27 participants (62.8%) consumed less than 50 g/day of fat, and 38 participants (88.4%) consumed less than 250 g/day of carbohydrates, indicating that many did not reach the reference values for these macronutrients.

### Nutrient and food intake patterns by energy intake adequacy

The median intake of the three major macronutrients and minerals was higher in the adequate/excess energy intake group than in the inadequate energy intake group, although these differences were not statistically significant (p>0.05) (Table 2). In contrast, for vitamins, no significant differences were observed for vitamin D or vitamin B_12_, whereas significant differences between the inadequate and adequate/excess energy intake groups were found for other vitamins (p<0.05) (Table 2). Furthermore, among participants whose intakes of the three major macronutrients were below the reference values, those in the inadequate energy intake group tended to have a higher intake of low-fat milk, canned tuna, tofu and deep-fried tofu, root vegetables, mushrooms, black and oolong tea, sugar, deep-fried foods, citrus fruits, persimmons, and strawberries than those in the adequate/excess energy intake group.

### Nutrient intake patterns by BMI category

For reference, Supplementary Table S1 presents the nutrient intake patterns for the two BMI-based groups (underweight, n=7; normal weight, n=41), showing no statistically significant differences in energy, macronutrient, mineral, vitamin, or other nutrient intake (p>0.05).

## Discussion

In this study, the dietary habits and nutritional intake of female university students were assessed using the BDHQ, and nutrient intake patterns were examined in relation to BMI categories and differences in energy intake.

Previous large-scale surveys among Japanese female university students have shown markedly unbalanced intake patterns, such as low carbohydrate and high fat intake in obese groups, whereas clear differences in nutrient intake across BMI categories are often difficult to detect, likely due to substantial inter-individual variability and the influence of background factors.^8^ Consistent with these findings, this study did not find statistically significant differences in energy or macronutrient intake between underweight and normal-weight participants. However, by focusing not only on BMI categories but also on the adequacy of estimated energy requirements, this study analyzed the nutritional status of female university students in the context of diverse lifestyles and food choices that cannot be explained by body size alone.

In the analyses based on energy intake adequacy, the inadequate energy intake group showed a particularly pronounced insufficiency in carbohydrate intake, with 88.4% of participants consuming less than the reference value. This suggests a tendency toward low-carbohydrate preference and avoidance of staple foods. The high proportion of participants whose intake of the three major macronutrients was below the reference values indicates qualitative issues in the diets of female university students. These nutrient intake characteristics cannot be fully captured by BMI-based body size classifications alone and were revealed by focusing on the discrepancy between actual energy intake and individual estimated energy requirements.

In terms of anemia-related nutrients (iron, zinc, vitamin B_12_, vitamin C, and folate), no clear group differences were observed according to the BMI category, whereas the group with energy intake below the estimated requirement showed marked inadequacies in these nutrients. Students should consciously select foods rich in protein, iron, folate, and other essential nutrients, and nutritional assessments should consider the overall balance between energy intake and individual energy requirements.

In terms of food group intake, the inadequate energy intake group consumed more low-fat milk, soybean products, root vegetables, mushrooms, and fruits, while also consuming high-fat and high-sugar foods, such as deep-fried foods and sugar. This pattern suggests that the coexistence of health-conscious food choices with the intake of snacks and highly palatable foods reflects contradictory eating behaviors. Such behaviors may be influenced by social stress and daily psychological burden, which can increase the desire for high-fat and high-sugar foods.^13^

Classification based on estimated energy requirements revealed that female university students whose energy intake did not meet their estimated needs had an insufficient intake of many nutrients, including the three major macronutrients, and may be at an increased risk of anemia. Previous studies have reported associations between inadequate intake of iron, protein, and folate and the risk of anemia among young and underweight pregnant women,^14,15^ and the present findings are consistent with these reports. Future dietary guidance should address not only the quantity of food intake, but also dietary quality, diversity of food choices, individual living environments, and psychological factors.

This study had several limitations. This was a cross-sectional survey of female nursing students at a single university with a relatively small sample size and may therefore be subject to selection bias. In addition, the use of a self-administered BDHQ may have introduced reporting bias, and basic attributes, such as living arrangements (living with family versus living alone) and meal frequency (e.g., skipping breakfast), were not sufficiently evaluated. Detailed assessments of body composition, physical activity, and psychosocial factors were not performed. Future studies should include longitudinal studies in diverse populations, incorporate objective methods of nutritional assessment, and adopt multidimensional analyses encompassing both psychosocial and environmental factors. An accurate understanding of the nutritional status of female university students can be achieved by addressing these issues, thereby facilitating the development of concrete dietary guidance strategies that contribute to improved health and pregnancy outcomes.

## Conclusion

In this study, no statistically significant differences in nutrient intake were observed between BMI-based groups, whereas clear differences were observed between the inadequate and adequate/excess energy intake groups. In the inadequate energy intake group, 88.4% had insufficient carbohydrate intake, and deficiencies were evident for the three major macronutrients and key nutrients, such as iron and folate. Furthermore, a complex eating pattern was observed in which health-conscious and high-calorie foods coexisted.

These findings indicate that the nutritional assessment of female university students should not rely solely on BMI but should instead adopt a multidimensional approach that considers individual eating behaviors, living environments, and psychological factors. Future studies should develop and implement specific and individualized nutrition counseling strategies tailored to each student.

## Figures and Tables

**Table 1:** Baseline characteristics by energy intake adequacy group

Variable	Inadequate energy intake group (n=43)	Adequate/excess energy intake group (n=7)	p-value^†^
Age (years)	19.0 [19.0–21.0]	19.0 [19.0–19.0]	0.476 (n.s.)
Height (cm)	158.0 [154.0–162.0]	161.0 [156.0–165.0]	0.364 (n.s.)
Weight (kg)	53.0 [47.5–56.2]	52.0 [48.0–52.6]	0.622 (n.s.)
BMI (kg/m^2^)	20.6 [19.5–22.2]	20.0 [19.0–20.4]	0.193 (n.s.)

The groups were classified based on whether their daily energy intake met the estimated energy requirements calculated for each participant. Data are presented as the median [interquartile range]. n.s.=not significant (p≥0.05); ^†^ Mann-Whitney U test.

**Table 2:** Comparison of estimated energy requirements and nutrient intake by energy intake adequacy group

Variable	Inadequate energy intake group (n=43)	Adequate/excess energy intake group (n=7)	p-value^†^
BMI (kg/m^2^)	20.6 [19.5–22.2]	20.0 [19.0–20.4]	0.193 (n.s.)
Protein (g/day)	52.1 [42.4–59.7]	81.2 [67.0–84.2]	0.000 (**)
Fat (g/day)	44.2 [38.3–56.4]	73.2 [66.6–81.9]	0.000 (**)
Carbohydrates (g/day)	179.9 [136.9–225.2]	320.9 [311.9–469.8]	0.000 (**)
Sodium (mg/day)	2908.3 [2593.9–3763.3]	4897.9 [4042.2–5785.4]	0.000 (*)
Potassium (mg/day)	1649.2 [1401.0–2347.5]	2489.5 [2253.7–3799.9]	0.001 (**)
Calcium (mg/day)	351.4[269.5–465.8]	504.4 [410.2–601.6]	0.014 (*)
Magnesium (mg/day)	162.7[128.0–208.5]	244.2 [231.5–290.9]	0.000 (**)
Iron (mg/day)	5.6[4.5–7.6]	8.6 [8.0–10.5]	0.001 (**)
Zinc (mg/day)	6.3[4.6–7.5]	9.9 [8.3–10.3]	0.000 (**)
Copper (mg/day)	0.8[0.6–1.0]	1.3 [1.2–1.7]	0.000 (**)
Vitamin D (μg/day)	6.2[4.7–10.7]	6.8 [5.0–11.6]	0.763 (n.s.)
Vitamin B1 (mg/day)	0.6[0.5–0.7]	0.8[0.7–1.0]	0.000 (**)
Vitamin B12 (μg/day)	4.6[4.0–7.8]	5.1[4.8–9.8]	0.379 (n.s.)
Vitamin C (mg/day)	77.7[59.1–113.4]	119.0[80.7–223.1]	0.013 (*)
Folic acid (μg/day)	219.9[186.1–335.9]	365.6[325.5–508.0]	0.016 (*)
Saturated fatty acids (g/day)	12.5 [10.5–15.7]	21.7[19.1–23.3]	0.000 (**)
Monounsaturated fatty acids (g/day)	16.5[12.9–20.1]	26.7[24.9–28.9]	0.000 (**)
Polyunsaturated fatty acids (g/day)	11.0[8.7–13.0]	15.2[12.9–19.1]	0.002 (**)
Total dietary fiber (g/day)	8.0[5.7–10.7]	14.4 [9.6–21.0]	0.002 (**)
Salt equivalent (g/day)	7.3[6.5–9.5]	12.5 [10.3–14.6]	0.000 (**)
Sucrose (g/day)	9.2[4.7–12.2]	34.3 [12.9–36.1]	0.001 (**)

The groups were classified based on whether their daily energy intake met the estimated energy requirements calculated for each participant. Data are presented as the median [interquartile range]. n.s.=not significant (p≥0.05); *: p<0.05; **: p<0.01; ^†^ Mann–Whitney U test.

## Supplementary Material

PDF-Japanese

## References

[1] Ministry of Health, Labour and Welfare. Reiwa 5 nen kokumin kenkou eiyo chousa kekka no gaiyou (Summary of the results of the National Health and Nutrition Survey 2023); 2024 (in Japanese). <https://www.mhlw.go.jp/content/10900000/001338334.pdf> (Accessed June 28, 2025)

[2] Murofushi Y, Yamaguchi S, Kadoya H, Otsuka H, Ogura K, Kaga H, Yoshizawa Y, Tamura Y. Multidimensional background examination of young underweight Japanese women: focusing on their dieting experiences. Front Public Health 2023; 11: 1130252. 37333534 10.3389/fpubh.2023.1130252 PMC10273403

[3] Mori N, Asakura K, Sasaki S. Differential dietary habits among 570 young underweight Japanese women with and without a desire for thinness: a comparison with normal weight counterparts. Asia Pac J Clin Nutr 2016; 25: 97–107. 26965768 10.6133/apjcn.2016.25.2.04

[4] Iizuka K, Sato H, Kobae K, Yanagi K, Yamada Y, Ushiroda C, Hirano K, Ichimaru S, Seino Y, Ito A, Suzuki A, Saitoh E, Naruse H. Young Japanese Underweight Women with “Cinderella Weight” Are Prone to Malnutrition, including Vitamin Deficiencies. Nutrients 2023; 15: 2216. 37409654 10.3390/nu15092216 PMC10181057

[5] Marangoni F, Cetin I, Verduci E, Canzone G, Giovannini M, Scollo P, Corsello G, Poli A. Maternal Diet and Nutrient Requirements in Pregnancy and Breastfeeding. An Italian Consensus Document. Nutrients 2016; 8: 629. 27754423 10.3390/nu8100629 PMC5084016

[6] Park JS. Maternal and paternal nutrition before conception. J Korean Med Assoc 2011; 54: 818–824.

[7] Bazinenkov AM. Preconception Care. In: Obstetric Evidence Based Guidelines. 4th ed. Boca Raton: CRC Press; 2022: 1–14.

[8] Mehta, M, Izurieta, R, Nishio, A, Horita, R, & Yamamoto, M. Nutritional intake and metabolic parameters of Japanese university students with and without obesity: Sex-specific differences. PLoS One 2023; 18: e0285088. 37134079 10.1371/journal.pone.0285088 PMC10155953

[9] Japan Society for the Study of Obesity. Guidelines for the management of obesity disease. Tokyo: Life Science Publishing; 2022 (in Japanese).

[10] Kobayashi S, Murakami K, Sasaki S, Okubo H, Hirota N, Notsu A, Fukui M, Date C. Comparison of relative validity of food group intakes estimated by comprehensive and brief-type self-administered diet history questionnaires against 16 d dietary records in Japanese adults. Public Health Nutr 2011; 14: 1200–1211. 21477414 10.1017/S1368980011000504

[11] Sakata S, Tsuchihashi T, Oniki H, Tominaga M, Arakawa K, Sakaki M, Kitazono T. Relationship between salt intake as estimated by a brief self-administered diet-history questionnaire (BDHQ) and 24-h urinary salt excretion in hypertensive patients. Hypertens Res 2015; 38: 560–563. 25787036 10.1038/hr.2015.35

[12] Ministry of Health, Labour and Welfare. Nihonjin no shokuji sesshu kijun 2020 nenban (Dietary Reference Intakes for Japanese 2020); 2020 (in Japanese). <https://www.mhlw.go.jp/stf/seisakunitsuite/bunya/kenkou_iryou/kenkou/eiyou/syokuji_kijyun.html> (Accessed October 30, 2024)

[13] Hyldelund NB, Dalgaard VL, Byrne DV, Andersen BV. Why Being ‘Stressed’ Is ‘Desserts’ in Reverse—The Effect of Acute Psychosocial Stress on Food Pleasure and Food Choice. Foods 2022; 11: 1756. 35741954 10.3390/foods11121756 PMC9222595

[14] Shinozaki N, Murakami K, Masayasu S, Sasaki S. Usual Nutrient Intake Distribution and Prevalence of Nutrient Intake Inadequacy among Japanese Children and Adults: A Nationwide Study Based on 8-Day Dietary Records. Nutrients 2023; 15: 5113. 38140372 10.3390/nu15245113 PMC10746136

[15] Uno K, Takemi Y, Hayashi F, Hosokawa M. Nutritional status and dietary intake among pregnant women in relation to pre-pregnancy body mass index in Japan. Nihon Koshu Eisei Zasshi 2016; 63: 738–749. 28100893 10.11236/jph.63.12_738

