# Supplementary material for: Preliminary investigation of nutritional intake among female university students using the brief-type self-administered diet history questionnaire (BDHQ)
Source: Fujita Med J. 2026 May 14;12(3):222–6. doi: 10.20407/fmj.2025-031 (PMC13433087; doi:10.20407/fmj.2025-031)
Supplement: Supplementary file 1 — PDF-Japanese [file fmj-12-222_s1.pdf]

タイトルページ

タイトル：

簡易型自記式食事歴法質問票を用いた女子大学生の栄養摂取状況に関する予備的検討

ランニングタイトル（140 字以内）：

BDHQ による女子大学生における栄養摂取状況

全著者（フルネーム）学位（MD，PhD 等），所属：

著者名・学位：Masato Sugiura, RN, PhD[1], Mikiko Shimizu, RN, PhD[1], Harumi Kato, RN, PhD[1], Keisuke Iwase, RN, MS[1], Hironori Tsuzuki, RN, MS[2], Qiongai Jin, RN, PhD[1], Yumi Akashi, MS[1], Mariko Umemura, RN, MS[1], Noriko Suzuki, RN, PhD[3], Tomoko Tanaka, RN, PhD[4], Yukie Kitamura, RN, PhD[5], Yuki Higashimoto, PhD, MT[6], Iku Fujiwara, RN, MS[1], and Naoki Yamamoto, PhD[7]

所属：

[1] Faculty of Nursing, Fujita Health University, School of Health Sciences, Toyoake, Aichi, Japan

[2] Department of Nursing, Toyohashi Sozo University, Toyohashi, Aichi Prefecture, Japan

[3] Department of Nursing Science, Kitasato University School of Health Science, Sagamihara, Kanagawa, Japan

[4] Faculty of Health Care and Nursing, Juntendo University, Urayasu, Chiba, Japan

[5] Department of Nursing, School of Nursing, Tokyo University of Information Sciences, Chiba, Chiba, Japan

[6] Department of Clinical Microbiology, Fujita Health University, School of Medical Sciences, Toyoake, Aichi, Japan

[7] Research Promotion Headquarters, Fujita Health University, Toyoake, Aichi, Japan

論文の種類：

Short Report 短報

Corresponding author，連絡先住所，電話番号・メールアドレス：

Masato Sugiura, RN, PhD,

Faculty of Nursing, Fujita Health University School of Health Sciences,

1-98 Dengakugakubo, Kutsukake-cho, Toyoake, Aichi 470-1192, Japan

Telephone: 0562-93-2598; E-mail: s-mas@fujita-hu.ac.jp

抄録:

目的：女子大学生における栄養摂取状況と食習慣を探索的に検討し，BMI 分類および推定エネルギー必要量の充足状況から主要栄養指標を予備的に比較・分析した。

方法：看護学科の女子大学生 50 名を対象に簡易型自記式食事歴法質問票（BDHQ）を用いて食生活調査を実施した。BMI 分類（やせ型群 7 名，普通型群 41 名）および推定エネルギー必要量（必要量未満群 43 名，必要量以上群 7 名）で群分けし，エネルギー必要量群間の栄養素摂取状況に着目し，BMI 分類を検討した。

結果：BMI 群間ではエネルギーや三大栄養素摂取量に有意差は認められなかった（$p>0.05$）。一方，エネルギー必要量群間では，必要量未満群で炭水化物摂取不足者が 88.4%で，たんぱく質，ビタミン C，鉄，葉酸の摂取量が有意に低値を示した（いずれも $p<0.05$）。

考察：女子大学生では，BMI による群間差はみられなかったが，エネルギー必要量未満群では炭水化物，たんぱく質，鉄，ビタミン C，葉酸など栄養素の摂取不足が顕著で，これは BMI のみでは捉えきれない食生活の問題を示しており，個別の栄養指導の必要性が示唆された。

結論：女子大学生の栄養評価には，BMI に加えエネルギー必要量充足度にも着目した個別対応が必要である。

キーワード:

女子大学生・簡易型自記式食事歴法質問票（BDHQ）・食習慣・栄養摂取状況・エネルギー必要量

緒　言

日本の 20～29 歳女性の低体重者の割合は 20.2%と高い[1]。この背景には，やせ志向やダイエット経験の多さ，運動習慣や体重認識の変化などがある[2]。やせ願望の有無で食習慣が異なり，低体重女性の約 90%で栄養素摂取量が不足し，葉酸やビタミン B_{12} の欠乏が顕著である[3]。こうした栄養不足は，妊娠前からのバランスの取れた食事の欠如と相まって，母体の健康や妊娠転帰に悪影響を及ぼすリスクが高い[4]。そのため，若年女性の早期からの栄養改善は社会的課題である。

特に女子大学生は，経済的・時間的制約や栄養知識の不足，やせ願望が重なり，炭水化物や必須栄養素の摂取が特に少ない低体重リスクの高い集団である[5]。この世代に対する妊娠前からの健康管理（プレコンセプションケア）の重要性は国際的に認識されている[6,7]。近年，日本の大学生を対象とした大規模研究では，BMI 分類による栄養摂取パターンの性差や傾向が報告されているものの[8]，個人差が大きく，女性集団では BMI 分類のみで栄養摂取の違いが必ずしも明確とはいえないことも示唆されている。実際，多様な生活背景やエネルギー必要量の違いが栄養状態に及ぼす影響の検証は十分ではない。

こうした背景をふまえ，本研究は女子大学生を対象に栄養摂取状況と食習慣を探索的に検討し，BMI 分類および推定エネルギー必要量の充足状況から主要な栄養指標を予備的に比較・分析することを目的とした。なお，BMI 分類に基づく比較では有意な差が見られなかったため，主な結果はエネルギー必要量充足別の比較に基づいて報告する。

研究方法

研究デザインと対象

本研究は横断的観察研究であり，2022 年 12 月～2023 年 2 月に愛知県内の私立 A 大学の看護学科に在籍する 1～4 年生の女子学生（約 500 名）を母集団とした。学内掲示による公募を行い，掲示を見て申し込みのあった学生 53 名に対し，研究説明・同意取得（書面および口頭）を行い，過去 1 か月の食生活について簡易型自記式食事歴法質問票（brief-type self-administered diet history

questionnaire ; BDHQ）を用いて食習慣調査を実施した。回答は自己記入式とし，BDHQ 記入時に身長・体重を各自が自己記載した値から BMI を算出した。収集データは業者が集計し，その後 Excel で整理して，記入漏れや明らかな誤記入のあるデータを除外し，最終的に 50 名分を解析対象とした。対象者の BMI は日本肥満学会基準（肥満症診療ガイドライン, 2022）[9] に基づき，18.5 未満を「やせ型」，18.5 以上 25.0 未満を「普通型」，25.0 以上 30.0 未満を「肥満 1 度」と分類した。BMI25.0 以上の者は肥満と判定され，やせ型・普通型とは栄養摂取傾向が大きく異なる可能性があるため，BMI による群分け（やせ型・普通型）による比較では除外した。一方，推定エネルギー必要量の充足状況に基づく群分け（必要量未満群・必要量以上群）による比較では，肥満 1 度の者を含む全対象者を解析に用いた。

BDHQ（簡易型自記式食事歴法質問票）の概要

本研究で使用した BDHQ は，A3 両面・平均回答時間約 15 分で，直近 1 か月間の食生活を簡便に評価できる質問紙である。本票は食品摂取頻度調査票 DHQ の簡易版として開発され，約 80 問・58 食品項目からエネルギーや栄養素・食品摂取量，定性的食行動指標を算出する [10]。日本人成人において大規模な疫学調査や保健指導等で広く用いられており，食事摂取状況の合理的評価が示された一方，食塩摂取など一部栄養素の推定精度には限界がある [10,11]。

分析方法

各群のエネルギー摂取量，三大栄養素（たんぱく質，脂質，炭水化物），ミネラル（ナトリウム，カリウム，カルシウム，マグネシウム，鉄，亜鉛，銅），ビタミン（ビタミン D，B_1，B_{12}，C，葉酸），その他（飽和脂肪酸，一価不飽和脂肪酸，多価不飽和脂肪酸，総食物繊維，食塩相当量，ショ糖）の摂取量について，中央値（四分位範囲 : IQR）を算出した。BMI により「やせ型」と「普通型」の 2 群の比較には，Mann-Whitney の U 検定を用いた。また，推定エネルギー必要量の充足状況に基づく群分け（「必要量未満群」と「必要量以上群」）の比較についても同様の検定を実施した。三大栄養素については日本人の食事摂取基準（2020 年版）[12] に基づき，たんぱく質 65g/日，脂質 50g/日，炭水化物 250g/日を基準値とし，基準値未満あるいは基準値以上で傾向を評価した。

統計解析はIBM SPSSを用い，Mann-WhitneyのU検定で2群間の差を検討し，$p < 0.05$を有意差とした。

倫理的配慮

本研究は藤田医科大学医学研究倫理審査委員会の承認（承認番号：HM18-156）を得て実施した。対象者には研究目的・方法・参加の任意性・個人情報保護・結果公表について書面と口頭で説明し，書面による同意を取得した。参加は任意であり，同意撤回も可能であることを説明した。データは匿名化し，研究目的以外には使用しないことを保証した。

研究結果

研究参加者の基本特性と群分け

研究参加者の流れは **Figure1** に示した。解析対象 50 名の年齢は 18～22 歳（中央値 19.0 歳［IQR19.0-21.0］），身長 148.8～171.5cm（中央値 158.0cm［IQR 154.0-162.0］），体重 38.5～68.0kg（中央値 52.5kg［IQR47.6-56.1］），BMI16.2～27.0（中央値 20.6［IQR19.4-21.9］）であった。**Table 1** にはエネルギー必要量群分け（「必要量未満群 43 名・必要量以上群 7 名」）による基本特性を示した。両群間で年齢，身長，体重，BMI に統計的有意差は認められなかった（$p>0.05$）。

エネルギー必要量と栄養摂取状況

Table2 に，推定エネルギー必要量と摂取量との比較から分類した「必要量未満群（43 名）」と「必要量以上群（7 名）」の栄養摂取状況を示した。必要量未満群では，たんぱく質 65g 未満が 37 名（86.0%），脂質 50g 未満が 27 名（62.8%），炭水化物 250g 未満が 38 名（88.4%）と，多くが基準値を下回っていた。

エネルギー必要量別の栄養素・食品摂取傾向

三大栄養素およびミネラルについては，有意差は認められなかったものの，すべての項目で必要量以上群が必要量未満群より高値を示した（$p > 0.05$）（**Table2**）。一方，ビタミン類では，BMI およびビタミン D，ビタミン B_{12} では有意差が認められなかったが，それ以外のビタミン類では

必要未満群と必要以上群の間に有意差が認められた（$p<0.05$）（**Table2**）。さらに，三大栄養素摂取が基準値未満の個体群において，必要量未満群は必要量以上群に比べ，低脂肪乳，ツナ缶，豆腐・油揚げ，根菜，きのこ，紅茶・ウーロン茶，砂糖，揚げ物，柑橘類，かき，いちごなどの摂取が多い傾向がみられた。

BMI 分類による栄養摂取傾向の検討

参考として，BMI による 2 群（やせ型 7 名，普通型 41 名）での栄養摂取傾向を Supplementary **Table S 1** に示した。エネルギー摂取量，三大栄養素，ミネラル，ビタミン，その他栄養素の摂取量について群間比較を行ったが，統計的有意差は認められなかった（$p>0.05$）。

考　察

本研究では，女子大学生を対象に BDHQ を用いて食習慣と栄養摂取状況を分析し，BMI やエネルギー摂取量の違いによる栄養摂取傾向を検討した。

これまでの大規模調査でも，日本人女子大学生においては肥満群で低炭水化物・高脂肪といった摂取バランスの著しい偏りが認められている一方，BMI 群間での栄養素摂取量の明確な差は得られにくく，個人差や背景要因の影響が大きいことが指摘されている[8]。本研究でも同様に，やせ型と普通型の間でエネルギー摂取量や三大栄養素摂取量に統計的有意差は認められなかった。しかし，本研究では BMI 分類だけでなく推定エネルギー必要量の充足状況に着目することで，体型だけでは説明できない生活習慣や食の選択の多様性から女子大学生の栄養摂取状況を分析した。

エネルギー必要量群分けによる解析では，必要量未満群において特に炭水化物の摂取不足が顕著であり（必要量未満群の 88.4%が基準値未満），低糖質志向や主食回避の傾向が背景にあると考えられる。三大栄養素の摂取が基準値未満の者が多いことは，女子大学生における食事の質的な課題を示唆する。このような栄養摂取の実態は，BMI という体型分類だけでは捉えきれない特徴であり，実際の摂取量と個人の必要量との差に着目することで明らかになったと考えられる。

また，貧血関連栄養素（鉄，亜鉛，ビタミン B_{12}，ビタミン C，葉酸）については，BMI による

明確な群間差は認められなかったが，エネルギー摂取量が推定エネルギー必要量未満の群では，これら栄養素の摂取不足が顕著であった。たんぱく質源や鉄分，葉酸などの必須栄養素を含む食品を意識的に選択することが重要であり，エネルギー摂取量と個人の必要量とのバランスを総合的に評価する必要がある。

食品群別摂取状況では，エネルギー摂取量が不足している群で低脂肪乳や大豆製品，根菜，きのこ，果物などの摂取が多い一方，揚げ物や砂糖など高脂質・高糖質食品の摂取もみられた。これは，健康志向の食品選択と間食や嗜好品摂取が併存する矛盾した食行動の存在を示しており，社会的ストレスや日常的な精神的負担が高脂肪・高糖質食品への欲求を高める可能性が示唆される[13]。

エネルギー必要量を基準とした群分けにより，エネルギー摂取量が推定エネルギー必要量を下回る女子大学生では，三大栄養素を含む多くの栄養素の摂取不足が明らかとなり，貧血リスクの高さが示唆された。若年女性一般や低体重妊婦における鉄・たんぱく質・葉酸の摂取不足と貧血リスクが関連することは先行研究で報告されており[14, 15]，本研究の結果もこれを裏付けるものであった。今後は，食事の量だけでなく質や食品選択の多様性，個々の生活背景や心理的要因も考慮した食事指導が求められる。

本研究は，特定の大学の看護学科女子学生を対象とした横断的調査であり，サンプルサイズの小ささ，自己記入式 BDHQ によるバイアス，居住環境（実家・下宿の別）や食事回数（朝食欠食の有無）といった基本属性の評価不足，身体組成や身体活動量，心理社会的要因などの詳細な評価は行っていない。今後は，より多様な集団を対象とした縦断的研究や，客観的な栄養評価手法の導入，心理社会的・環境要因を含めた多面的な分析が必要である。こうした課題に取り組むことで，女子大学生の栄養状態の実態把握と，将来の健康や妊娠転帰の改善につながる具体的な食事指導方策の構築が期待される。

結　論

本研究により，女子大学生における栄養摂取状況の評価において，BMI 分類による群間では統計的有意差は認められなかったが，エネルギー必要量の充足状況による群分けでは明確な違いが確認された。エネルギー必要量未満群では炭水化物摂取不足が 88.4%と高率であり，三大栄養素や鉄，葉酸などの重要な栄養素の摂取不足が認められた。また，健康志向食品と高カロリー食品の摂取が併存する複雑な食行動パターンが確認された。

女子大学生の栄養状態評価においては, BMI のみに依存せず, 個々の食行動の特性や生活背景, 心理的要因を考慮した多面的な評価が重要である。今後は一人ひとりに応じた具体的な栄養指導の検討が必要である。

利益相反

研究者に，本研究に係わる開示すべき利益相反はない。

IRB 承認コードと機関名

本研究は，藤田医科大学医学研究倫理審査委員会の承認を得て実施した（承認番号 : HM18-156）。

患者/参加者からの出版同意（資料の取り扱い）

対象者には研究目的・方法を説明し，書面による同意を得た。収集したデータは匿名化して処理し，個人が特定されない形でのみ公表した。

資金提供

本研究は，科学研究費補助金 基盤研究(C) （課題番号 : 24K13873） の助成を受けて実施した。

謝　辞

本研究の実施にあたり，データ収集に協力いただいた私立 A 大学看護学科の学生の皆様に感謝

申し上げます。

【文　献】

1. Ministry of Health, Labour and Welfare. Reiwa 5 nen kokumin kenkou eiyo chousa kekka no gaiyou (Summary of the results of the National Health and Nutrition Survey 2023) ; 2024 (in Japanese). <https://www.mhlw.go.jp/content/10900000/001338334.pdf>（Accessed June 28, 2025）
2. Murofushi Y, Yamaguchi S, Kadoya H, Otsuka H, Ogura K, Kaga H, Yoshizawa Y, Tamura Y. Multidimensional background examination of young underweight Japanese women: focusing on their dieting experiences. Front Public Health 2023; 11: 1130252.
3. Mori N, Asakura K, Sasaki S. Differential dietary habits among 570 young underweight Japanese women with and without a desire for thinness: a comparison with normal weight counterparts. Asia Pac J Clin Nutr 2016; 25: 97-107.
4. Iizuka K, Sato H, Kobae K, Yanagi K, Yamada Y, Ushiroda C, Hirano K, Ichimaru S, Seino Y, Ito A, Suzuki A, Saitoh E, Naruse H. Young Japanese Underweight Women with "Cinderella Weight" Are Prone to Malnutrition, including Vitamin Deficiencies. Nutrients 2023; 15: 2216.
5. Marangoni F, Cetin I, Verduci E, Canzone G, Giovannini M, Scollo P, Corsello G, Poli A. Maternal Diet and Nutrient Requirements in Pregnancy and Breastfeeding. An Italian Consensus Document. Nutrients 2016; 8: 629.
6. Park JS. Maternal and paternal nutrition before conception. J Korean Med Assoc.2011; 54: 818-24.
7. Bazinenkov AM. Preconception Care. In: Obstetric Evidence Based Guidelines. 4th ed. Boca Raton: CRC Press; 2022: 1-14.
8. Mehta, M., Izurieta, R., Nishio, A., Horita, R., & Yamamoto, M. (2023). Nutritional intake and metabolic parameters of Japanese university students with and without obesity: Sex-specific differences. PLoS One, 18(5), e0285088. https://doi.org/10.1371/journal.pone.0285088

9. Japan Society for the Study of Obesity. Guidelines for the management of obesity disease. Tokyo: Life Science Publishing; 2022 (in Japanese).

10. Kobayashi S, Murakami K, Sasaki S, Okubo H, Hirota N, Notsu A, Fukui M, Date C. Comparison of relative validity of food group intakes estimated by comprehensive and brief-type self-administered diet history questionnaires against 16 d dietary records in Japanese adults. Public Health Nutr 2011; 14: 1200-11.

11. Sakata S, Tsuchihashi T, Oniki H, Tominaga M, Arakawa K, Sakaki M, Kitazono T. Relationship between salt intake as estimated by a brief self-administered diet-history questionnaire (BDHQ) and 24-h urinary salt excretion in hypertensive patients. Hypertens Res 2015; 38: 560-3.

12. Ministry of Health, Labour and Welfare. Nihonjin no shokuji sesshu kijun 2020 nenban (Dietary Reference Intakes for Japanese 2020); 2020 (in Japanese). <https://www.mhlw.go.jp/stf/seisakunitsuite/bunya/kenkou_iryou/kenkou/eiyou/syokuji_kijyun.html> （Accessed October 30, 2024）

13. Hyldelund NB, Dalgaard VL, Byrne DV, Andersen BV. Why Being 'Stressed' Is 'Desserts' in Reverse—The Effect of Acute Psychosocial Stress on Food Pleasure and Food Choice. Foods 2022; 11: 1756.

14. Shinozaki N, Murakami K, Masayasu S, Sasaki S. Usual Nutrient Intake Distribution and Prevalence of Nutrient Intake Inadequacy among Japanese Children and Adults: A Nationwide Study Based on 8-Day Dietary Records. Nutrients 2023; 15: 5113.

15. Uno K, Takemi Y, Hayashi F, Hosokawa M. Nutritional status and dietary intake among pregnant women in relation to pre-pregnancy body mass index in Japan. Jpn. J. Public Health 2016; 63: 738-49.

Figure Caption

Figure 1. Flow diagram of participant recruitment, eligibility assessment, exclusion, grouping, and analysis. Approximately 500 female nursing students (grades 1–4) were invited at A University, 53 consented to participate. After excluding three with incomplete/invalid responses, 50 patients were analyzed. The participants were classified according to energy intake adequacy (inadequate: n=43; adequate/excess: n=7) and BMI (underweight: n=7; normal weight: n=41). Two participants with grade 1 obesity (BMI ≥25.0) were excluded from BMI comparisons but included in energy intake analyses.

Tables

Table1. Baseline characteristics by energy intake adequacy group

Table2. Comparison of estimated energy requirements and nutrient intake by energy intake adequacy group

Table S１. Nutritional intake patterns by two BMI groups

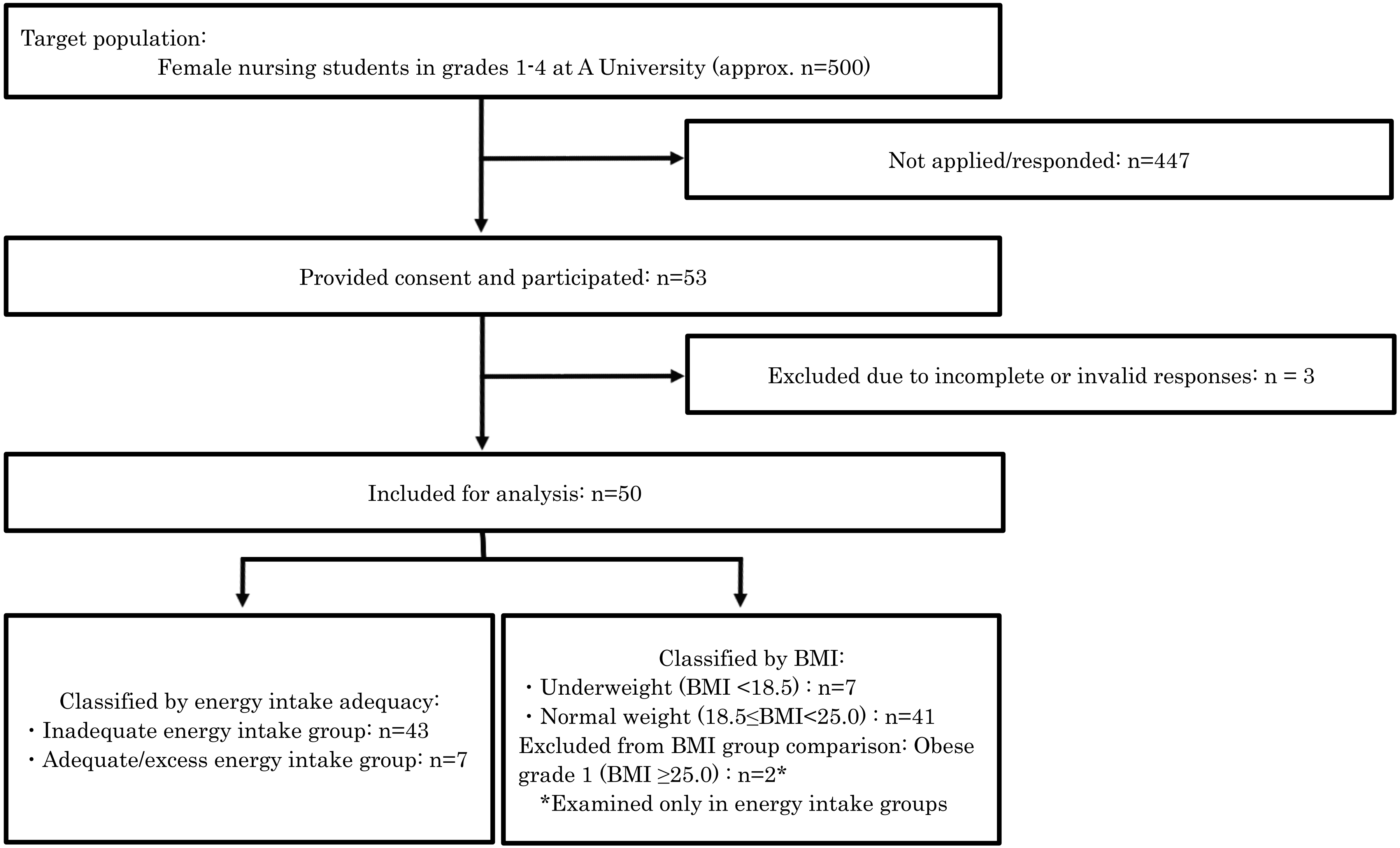

Figure 1. **Flow diagram of participant recruitment, eligibility assessment, exclusion, grouping, and analysis.**

Table 1. Baseline characteristics by energy intake adequacy group

Variable	Inadequate energy intake group (n=43)	Adequate/excess energy intake group (n=7)	p-value†
Age (years)	19.0 [19.0-21.0]	19.0 [19.0-19.0]	0.476（n.s.）
Height (cm)	158.0 [154.0-162.0]	161.0 [156.0-165.0]	0.364（n.s.）
Weight (kg)	53.0 [47.5-56.2]	52.0 [48.0-52.6]	0.622（n.s.）
BMI（kg/m^2）	20.6 [19.5-22.2]	20.0 [19.0-20.4]	0.193（n.s.）

The groups were classified based on whether their daily energy intake met the estimated energy requirements calculated for each participant.

Data are presented as the median [interquartile range]. n.s. = not significant ($p \geq 0.05$); †Mann-Whitney U test.

Table 2. Comparison of estimated energy requirements and nutrient intake by energy intake adequacy group

Variable	Inadequate energy intake group (n=43)	Adequate/excess energy intake group (n=7)	p-value†
BMI（kg/m^2）	20.6 [19.5-22.2]	20.0 [19.0-20.4]	0.193（n.s.）
Protein（g/day）	52.1 [42.4-59.7]	81.2 [67.0-84.2]	0.000（**）
Fat（g/day）	44.2 [38.3-56.4]	73.2 [66.6-81.9]	0.000（**）
Carbohydrates（g/day）	179.9 [136.9-225.2]	320.9 [311.9-469.8]	0.000（**）
Sodium（mg/day）	2908.3 [2593.9-3763.3]	4897.9 [4042.2-5785.4]	0.000（*）
Potassium（mg/day）	1649.2 [1401.0-2347.5]	2489.5 [2253.7-3799.9]	0.001（**）
Calcium（mg/day）	351.4[269.5-465.8]	504.4 [410.2-601.6]	0.014（*）
Magnesium（mg/day）	162.7[128.0-208.5]	244.2 [231.5-290.9]	0.000（**）
Iron（mg/day）	5.6[4.5-7.6]	8.6 [8.0-10.5]	0.001（**）
Zinc（mg/day）	6.3[4.6-7.5]	9.9 [8.3-10.3]	0.000（**）
Copper（mg/day）	0.8[0.6-1.0]	1.3 [1.2-1.7]	0.000（**）
Vitamin D（μg/day）	6.2[4.7-10.7]	6.8 [5.0-11.6]	0.763（n.s.）
Vitamin B1（mg/day）	0.6[0.5-0.7]	0.8[0.7-1.0]	0.000（**）
Vitamin B12（μg/day）	4.6[4.0-7.8]	5.1[4.8-9.8]	0.379（n.s.）
Vitamin C（mg/day）	77.7[59.1-113.4]	119.0[80.7-223.1]	0.013（*）
Folic acid（μg/day）	219.9[186.1-335.9]	365.6[325.5-508.0]	0.016（*）
Saturated fatty acids（g/day）	12.5 [10.5-15.7]	21.7[19.1-23.3]	0.000（**）
Monounsaturated fatty acids（g/day）	16.5[12.9-20.1]	26.7[24.9-28.9]	0.000（**）
Polyunsaturated fatty acids（g/day）	11.0[8.7-13.0]	15.2[12.9-19.1]	0.002（**）
Total dietary fiber（g/day）	8.0[5.7-10.7]	14.4 [9.6-21.0]	0.002（**）
Salt equivalent（g/day）	7.3[6.5-9.5]	12.5 [10.3-14.6]	0.000（**）
Sucrose（g/day）	9.2[4.7-12.2]	34.3 [12.9-36.1]	0.001（**）

The groups were classified based on whether their daily energy intake met the estimated energy requirements calculated for each participant.

Data are presented as the median [interquartile range]. n.s.= not significant ($p \geq 0.05$); *: $p < 0.05$; **: $p < 0.01$; †Mann–Whitney U test.
