# Supplementary material for: Preliminary investigation of nutritional intake among female university students using the brief-type self-administered diet history questionnaire (BDHQ)
Source: Fujita Med J. 2026 May 14;12(3):222–6. doi: 10.20407/fmj.2025-031 (PMC13433087; doi:10.20407/fmj.2025-031)
Supplement: Supplementary file 2 — Supplementary Tables [file fmj-12-222_s2.pdf]

**Supplementary Table S１． Nutritional intake patterns by two BMI groups**

| Variable | Underweight（n=7） | Normal weight（n=41） | p-value† |
|---|---|---|---|
| Energy（kcal/day） | 1374.7 [1177.6-2091.7] | 1468.5 [1095.6-1866.9] | .864（n.s.） |
| Protein（g/day） | 52.8 [45.5-71.4] | 54.0 [42.7-64.3] | .627（n.s.） |
| Fat（g/day） | 52.0 [43.3-70.5] | 46.3 [38.8-61.5] | .474（n.s.） |
| Carbohydrates（g/day） | 179.9 [150.2-266.6] | 196.9 [137.5-248.2] | .932（n.s.） |
| Sodium（mg/day/day） | 2785.5 [2593.9-4229.8] | 3265.5 [2724.6-4173.6] | .775（n.s.） |
| Potassium（mg/day） | 1861.2 [1572.3-3009.3] | 1779.7 [1450.4-2417.7] | .510（n.s.） |
| Calcium（mg/day） | 480.8 [297.3-556.0] | 409.7 [277.6-492.1] | .300（n.s.） |
| Magnesium（mg/day） | 164.9 [150.4-275.4] | 170.0 [134.3-233.8] | .627（n.s.） |
| Iron（mg/day） | 5.6 [4.7-10.6] | 6.5 [4.5-8.1] | .529（n.s.） |
| Zinc（mg/day） | 6.7 [5.5-8.8] | 6.5 [4.8-8.1] | .567（n.s.） |
| Copper（mg/day） | 0.9 [0.6-1.3] | 0.9 [0.7-1.2] | .567（n.s.） |
| Vitamin D（μg/day/day） | 6.6 [4.6-10.7] | 6.3 [4.8-11.3] | .886（n.s.） |
| Vitamin B１（mg/day） | 0.6 [0.5-0.8] | 0.6 [0.5-0.7] | .567（n.s.） |
| Vitamin B12（μg/day） | 4.5 [4.0-7.7] | 4.9 [4.1-8.2] | .710（n.s.） |
| Vitamin C（mg/day） | 72.5 [59.1-135.7] | 84.4 [63.7-121.8] | .954（n.s.） |
| Folic acid（μg/day） | 222.5 [198.0-430.6] | 249.4 [184.9-384.7] | .567（n.s.） |
| Saturated fatty acids（g/day） | 13.1 [12.8-18.2] | 12.7 [10.4-17.7] | .274（n.s.） |
| Monounsaturated fatty acids（g/day） | 18.0 [15.7-26.7] | 17.4 [13.4-21.7] | .627（n.s.） |
| Polyunsaturated fatty acids（g/day） | 12.8 [9.5-16.4] | 11.4 [8.8-13.7] | .492（n.s.） |
| Total dietary fiber（g/day/day） | 9.3 [7.7-16.3] | 8.6 [6.2-12.4] | .328（n.s.） |
| Salt equivalent（g/day） | 7.0 [6.5-10.7] | 8.2 [6.9-10.6] | .775（n.s.） |
| Sucrose（g/day） | 16.6 [3.4-18.2] | 10.0 [6.3-12.8] | .389（n.s.） |

Values are presented as median [interquartile range]. n.s.=not significant ($p \geq 0.05$); *: $p<0.05$; **: $p<0.01$; †Mann–Whitney U test.
